# A Resource Allocation Mechanism Based on Weighted Efficiency Interference-Aware for D2D Underlaid Communication

**DOI:** 10.3390/s19143194

**Published:** 2019-07-19

**Authors:** Jingzhao Li, Xiaoming Zhang, Yuan Feng, Kuan-Ching Li

**Affiliations:** 1 College of Electrical and Information Engineering, Anhui University of Science and Technology (AUST), Huainan 232001, China; 2 Department of Computer Science and Information Engineering, Providence University, Taichung 43301, Taiwan

**Keywords:** D2D communication, resource allocation, SINR, throughput

## Abstract

Device-to-device (D2D) communication is a promising technique for direct communication to enhance the performance of cellular networks. In order to improve the system throughput and utilization of spectrum resource, a resource allocation mechanism for D2D underlaid communication is proposed in this paper where D2D pairs reuse the resource blocks (RBs) of cellular uplink users, adopting a matching matrix to disclose the results of resource allocation. Details of the proposed resource allocation mechanism focused are listed as: the transmit power of D2D pairs are determined by themselves with the distributed power control method, and D2D pairs are assigned to different clusters that are the intended user sets of RBs, according to the threshold of the signal-to-interference-plus-noise ratio (SINR). The weighted efficiency interference-aware (WE-I-A) algorithm is proposed and applied subsequently to promote the system throughput by optimizing the matching of D2D pairs and RBs, where each D2D pair is weighted based on the SINR to compete for the priority of RBs fairly. Simulation results demonstrate that the proposed algorithm contributes to a good performance on the system throughput even if the uplink state is limited.

## 1. Introduction

D2D communication is a novel wireless communication technique where devices located nearby can communicate directly and remain controlled by the base station (BS) [1,2,3,4]. It is a crucial technology for LTE-A that remains good backward compatibility with LTE (TDD & FDD), which means the functions in LTE (TDD & FDD) can be available in LTE-A. D2D communication can work within two modes: overlaid and underlaid. The scenario discussed in this paper is underlaid mode where D2D pairs reuse the RBs of cellular users (CUs), greatly enhancing the spectral efficiency and relieve the load of BSs [5], in which energy consumption and clustering are the essential contents of research [6]. Under this communication method, devices transmit the data signals to associated receivers over direct links by reusing the cellular RBs [7] that is different from cellular communication where devices must communicate through a BS [8]. Thus, the spectral efficiency of a direct link is much higher due to resource sharing [9,10].

D2D communication underlaid cellular network enhances the performance of the network with much lower protocol overhead than existing cellular networks [11], such that D2D pairs with low transmit power reuse RBs of CUs to achieve high throughput and low delays, and decrease energy consumption [12,13]. Although D2D communication has received incremental attentions in both 4G and 5G networks [14], the performance of system throughput and spectral efficiency can be improved [15]. Meanwhile, the interference to the cellular network is inevitably enhanced due to RBs’ sharing [16]. The method adopted to deal with the interference problem when CUs share RBs with D2D pairs is depicted as: the power control procedure is implemented to ensure that the interference from D2D pairs to CUs do not exceed a given threshold, followed by the application of a proper allocation algorithm to optimize the matching of D2D pairs and RBs to achieve a significant system performance increase.

One of the effective methods to increase the performance of communication systems is to build more BSs [17]. However, such an act is impractical due to the factors as high cost, limited space, and low utilization ratio. There are several studies in the literature on resource allocation for D2D communication. In [18], authors tried to reduce the uplink interference in two steps for D2D communication, where CUs share RBs with D2D pairs under the condition that the interference between D2D pairs and CUs are permitted. Nevertheless, the proposed resource allocation method based on Kuhn–Munkres (KM) algorithm would not be effective when the number of D2D pairs and CUs is large. As an improvement, authors managed the interference by dynamic graph framework for multi-hop D2D communication, where the communication system consists of multiple nodes and D2D pairs [19]. Presented in [20,21], the auction-based method was used in resource allocation problems for the D2D network, where the BS is visualized as the auctioneer, the RBs as the goods, and the users as the bidders. This method could solve resource allocation issues well in a cellular network, despite it being a concept of business at the initial stage. A scenario of Orthogonal Frequency Division Multiple Access (OFDMA) network is depicted in [22], where the method of Lagrange decomposition is adapted to manage sub-channels and transmit power in the two-tier uplink network.

Power control is one of the effective methods to solve interference caused by D2D pairs to CUs in both uplink and downlink cases [23]. Huang et al. attempt to satisfy the required system throughput with power constraints of BS and minimized the users’ transmission power in a scenario of the multi-node wireless network [24]. However, it is difficult for the proposed algorithm to obtain the optimal solution in cases of nonlinear complexity systems. Formulated as an optimization issue under the constraints of transmitting power, maximization of system throughput is studied in [25] to provide a cooperative power allocation method. In [26], the energy harvesting relays are considered and then proposed an algorithm for user association, resource allocation, and power control in the heterogeneous multi-tier network. Also, the resource allocation with more realistic bursty traffic is optimized by a centralized resource allocation method with delay-aware and a power control framework that is based on queuing model [27]. Considering the abovementioned literature, a problem is that the fairness for wireless nodes or users has not been taken into consideration. The links with poor states may lose the ability of communication in the competition with good-state links, such a phenomenon known as ‘starvation’ in communication systems.

In this paper, we propose a low complexity and effective spectrum resource allocation mechanism for D2D communication based on the interference-aware method. In the uplink stage, the interference can be sensed by associated D2D receivers, as they are different due to diverse channel gains and transmit power. Therefore, D2D pairs with good uplink states may have priority to share RBs with CUs, which is unfair for poor D2D link. In regard to this problem, we weight the D2D pairs and promote the efficiency of resource allocation. The contributions of this paper are shown as follows:

- The proposed resource allocation mechanism based on weighted efficiency interference-aware (WE-I-A) can guarantee the Quality-of-Service (QoS) of D2D pairs and CUs. On the premise of distributed power control method, we manage the interference and cluster the D2D pairs in a novel way that each D2D pair can be an intended user of different clusters in terms of the constraint of SINR. As defined in the proposed scheme, each D2D pair should be in the different clusters and each cluster can only remain one D2D pair after resource allocation,
- Due to ‘starvation’ of poor-state D2D link, we attempt to overcome the problem by weighting D2D pairs in the clusters with received SINR for fairness. By this way, each D2D pair competes to be the actual user of the cluster by weight value rather than that D2D pairs with good link states have priority to reuse RBs. The active D2D pairs can be increased while promoting the effectiveness of RBs. As a consequence, the overall performance of system throughput is highly enhanced.

The rest of this paper is organized as follows. The system model of the D2D underlaid cellular network, and the expression of the system throughput are presented in Section 2, and the resource allocation mechanism based on weighted efficiency interference-aware is proposed in Section 3. In Section 4, simulation results and relevant analysis of the SINR, throughput, and utility of allocation are depicted and discussed, and finally, concluding remarks, and future directions are presented in Section 5.

## 2. System Model

In this section, the system model of D2D communication is presented. The communication scenario of D2D pairs and CUs in a single cell is presented and discussed first, then the expression of system throughput is followed.

### 2.1. Scenario Description

**Figure 1:**
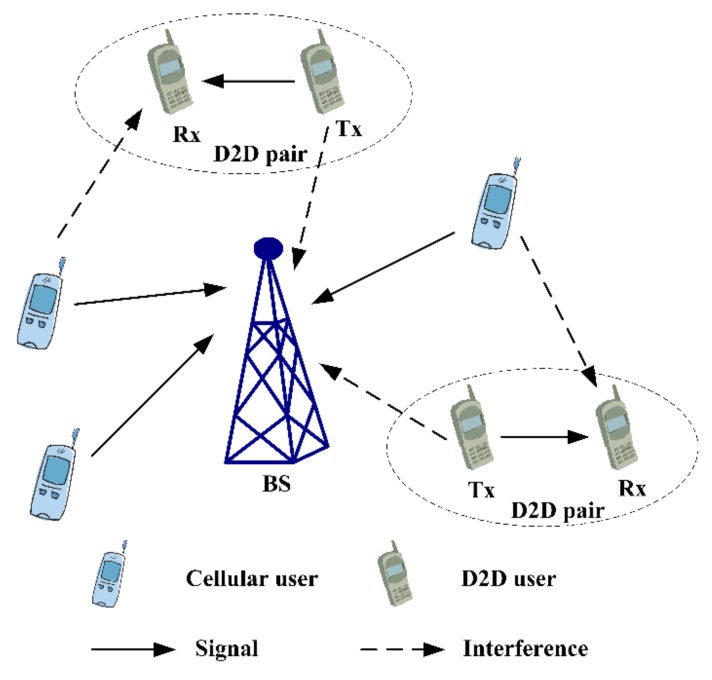
Scenario of D2D communication with uplink RBs’ reusing.

When the RBs are reused by D2D pairs in the downlink cellular network, the interference from CUs to D2D pairs can be inhibited by cascade precoding and beamforming. Meanwhile, it is difficult to analyze the interference caused by D2D pairs, as it affects all CUs in downlink cellular network. Additionally, the efficiency of spectrum resources in downlink is higher than that in the uplink. Consequently, the case that D2D pairs reuse the RBs of cellular uplink users is investigated in this research. As shown in Figure 1, D2D transmitters cause interference to cellular uplink users at BS, while cellular uplink users cause interference to D2D receivers mutually.

**Figure 2:**
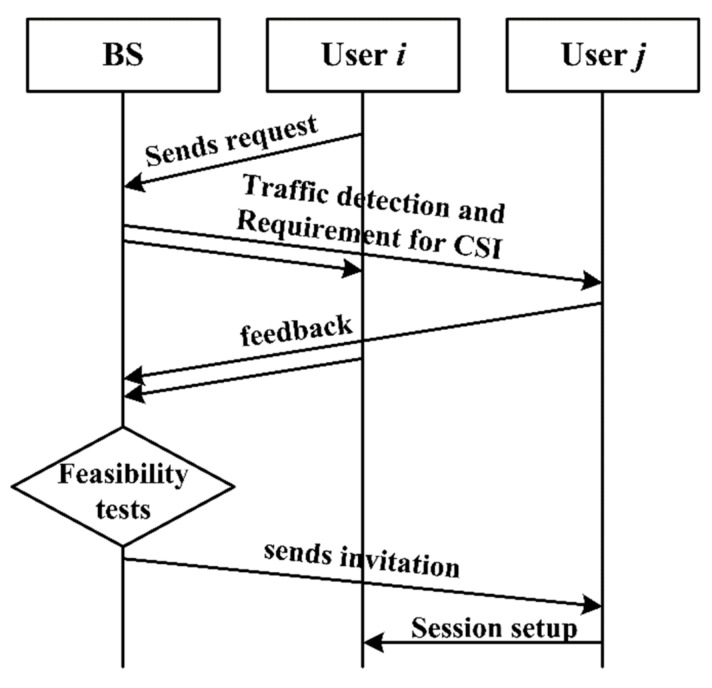
Session setup of D2D communication.

After the RBs are scheduled to cellular uplink users by BS, each CU can only occupy one RB in a scheduling period to transmit signals. That is, each CU corresponds to one RB, so the available RBs mentioned henceforth in the paper are those that have occupied by CUs. Then D2D communication is set up under this situation, as shown in Figure 2.

The steps of session setup for D2D communication are shown as:

(1) User *i* sends the request of D2D communication to BS.
(2) BS selects user *j* by traffic detection and then requires both users to feedback the Channel State Information (CSI).
(3) Feasibility tests for D2D communication are implemented by BS.
(4) If both users satisfy the conditions of session setup, BS send the invitation of user *i* to user *j*.
(5) D2D communication is set up between user *i* and user *j*, and a D2D pair is formed.

As shown in Figure 2, BS is vigorous to determine the session setup of D2D communication, as D2D pairs communicate while remaining detected by BS. That is, if two users are not proper to communicate in underlaid mode, both of them would be back to work in the overlaid mode which can refer to CUs communicating in cellular mode, users communicate through the usage of the orthogonal RBs appropriately allocated. In the underlaid mode, BS allocates the RBs of CUs to each D2D pair, whereas the transmit power is determined differently according to the diverse power control methods. The centralized power control is that the transmit power of D2D pairs are managed by BS, while D2D pairs determine the transmit power by themselves in a distributed power control method. In this paper, the latter is adopted.

### 2.2. System Throughput

**Figure 3:**
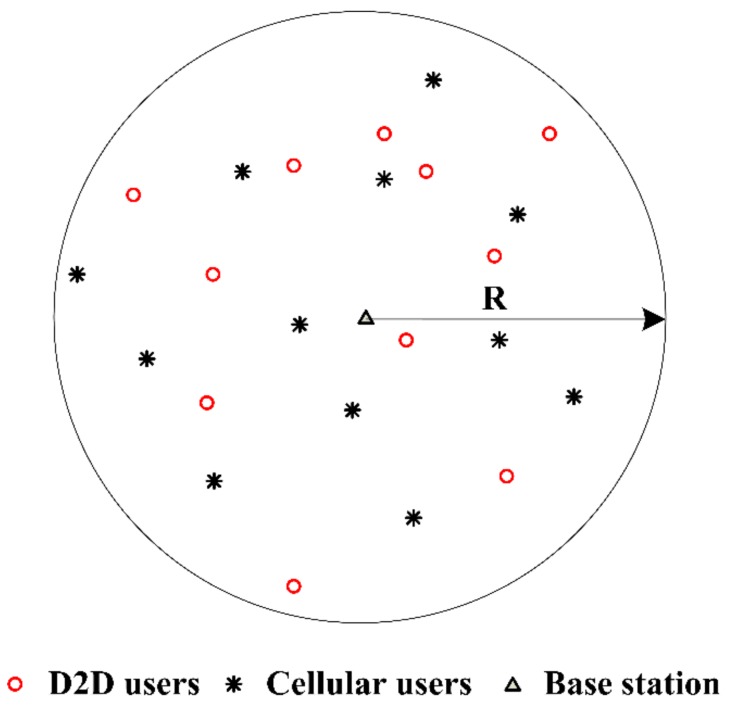
Illustration of a communication area where users are normally distributed around the base station.

D2D underlaid cellular network is considered to be located in a region with radius R, where BS is the center of this region as illustrated in Figure 3 (each D2D pair is described as a point due to the short distance between transmitter and receiver). D2D pairs and CUs are independent identically distributed (i.i.d.) around the BS within the region, and the positions of D2D receivers follow a uniform distribution inside the region that is close enough to satisfy the maximum distance constraints of D2D communication. Furthermore, the channel is modeled as the Rayleigh fading channel, and its response follows the i.i.d. complex Gaussian distribution.

As the system model is established independently in a single cell, the interference from other cells is ignored. D2D pairs and CUs interfere mutually due to the resource sharing in the same sub-channel. Hence, the total number of available RBs and D2D pairs in the communication region are assumed to be *M* and *N*, where *X* = {1, 2, 3, …, *M*} and *Y* = {1, 2, 3, …, *N*}, respectively. The set of RBs is denoted as $R\;=\;\left\{RB_{1},RB_{2},\ldots ,RB_{j},\ldots ,RB_{M}\right\}$ and let $DP\;=\;\left\{D2D_{1},\;D2D_{2},\ldots ,\;D2D_{i},\ldots ,\;D2D_{N},\right\}$ be the set of D2D pairs. In the uplink, the *j*-th CU transmits signal $s_{c}$ to the BS, and the *i*-th D2D pair reuses the same RB to transmit signal $s_{d}$. Here, a matching matrix is used to reveal the state of resource allocation.

 **Definition 1.**  *The resource sharing between D2D pairs and RBs is denoted as a matching matrix*$\beta^{CD}\;=\;{\left[\beta_{i,j}^{CD}\right]}_{N\times M}$, where $\beta_{i,j}^{CD}\in \left\{0\;,\;1\right\}$ is the binary decision variable to show the result of allocation as

(1) $$\beta_{i,j}^{CD}=\left\{\begin{array}{l} 1,\;\mathrm{if}\;D2D\_i\;\mathrm{reuse}\;\mathrm{RB}\_j\\ 0,\;\mathrm{otherwise}\; \end{array}\right.$$

Thus, the received signals at D2D receiver and BS are given respectively as

(2) $$y_{D\_i}=d_{i,i}^{-\frac{\alpha }{2}}h_{i,i}s_{d}+\beta_{i,j}^{CD}d_{j,i}^{-\frac{\alpha }{2}}h_{j,i}s_{c}+N_{d}$$

(3) $$y_{C\_j}=d_{j,i}^{-\frac{\alpha }{2}}h_{j,B}s_{c}+\beta_{i,j}^{CD}d_{i,B}^{-\frac{\alpha }{2}}h_{i,B}s_{d}+N_{c}$$

where $h_{x,y}$ represents the channel response of the *x–y* link that is from device *x* to *y*, and distributes independently as complex Gaussian distribution $\;CN\left(0,1\right)$. Hence, the path-loss model is assumed to be distance dependent and α is the path-loss exponent.$\;N_{d}$ and $N_{c}$ denote the additive noise at D2D receivers and the BS, which is distributed as $CN\left(0,\sigma^{2}\right)$. Finally, the distance from device x to y is denoted as $d_{x,y}$.

The signal-to-interference-plus-noise ratio (SINR) at D2D receivers are given by [28]

(4) $$\gamma_{D\_i}=\frac{p_{D\_i}d_{i,i}^{-\alpha }{\left|h_{i,i}\right|}^{2}}{I_{cd}^{i}+I_{dd}^{i}+N_{d}}$$

where $I_{cd}^{i}\;=\;\overset{M}{\underset{j=1}{\sum }}\beta_{i,j}^{CD}p_{C\_j}d_{j,i}^{-\alpha }{\left|h_{j,i}\right|}^{2}$ is the interference from CUs; $I_{dd}^{i}\;=\;\underset{k\in DU_{j},\;k\neq i}{\sum }p_{C\_k}d_{k,i}^{-\alpha }{\left|h_{k,i}\right|}^{2}$ denotes the interference from neighboring D2D pairs in the same sub-channel and $DU_{j}$ is D2D pairs in j-th sub-channel; D_i and C_j represent i-th D2D pair and j-th CU; $p_{D\_i}$ and $p_{C\_j}$ are the transmit power of i-th D2D pair and j-th CU, respectively. The CUs have no interference to each other since CUs communicate with orthogonal spectrum resources in the cellular system. Therefore, the received SINR of cellular uplink at BS is given by

(5) $$\gamma_{C\_j}=\frac{p_{C\_j}d_{j,B}^{-\alpha }|h_{j,B}{|}^{2}}{I_{dc}^{j}+N_{C}}$$

where $I_{dc}^{j}=\sum_{i=1}^{N}\beta_{i,j}^{CD}p_{D\_i}d_{i,B}^{-\alpha }|h_{i,B}{|}^{2}$. As stated, this research aims to maximize the system throughput under the condition of guaranteeing the reliable communication of CUs and D2D pairs. Thus, we establish the model of system throughput according to the Shannon capacity formula as

$$R^{B}=R_{D}+R_{C}$$

(6) $$=\sum_{i=1}^{N}B_{d}{log}_{2}\left(1+\gamma_{D\_i}\right)+\sum_{j=1}^{M}B_{d}{log}_{2}\left(1+\gamma_{C\_j}\right)$$

s.t.

(7) $$\gamma_{D\_i}\geq \gamma_{min}^{\left(D\right)},\forall \;i\in Y$$

(8) $$\gamma_{C\_j}\geq \gamma_{min}^{\left(C\right)},\forall j\in X$$

(9) $$p_{D\_i}\leq p_{D\_max,}\forall \;i\in Y$$

(10) $$p_{C\_j}\leq p_{C\_max,}\forall j\in X$$

where ***B*** denotes all possible allocation results;$B_{d}$ is the bandwidth of sub-channel; $R_{D}$ and $R_{C}$ are the throughput of D2D pairs and CUs, respectively. The constraints in (7) and (8) represent the low bound of SINR for D2D pairs and CUs respectively, while (9) and (10) ensure that the transmit power of D2D pairs and CUs are not permitted to exceed the maximum power limit.

The optimization problem indicated above is observed to be an NP-Hard problem. The exhaustive search method can perform well on this problem, while the complexity is high enough to limit the utilization in practice.

Presently, fairness has not been taken into account in existing literature, as [29,30]. The situation that one of the D2D pairs may communicate by using few RBs that tend to be occupied by other D2D pairs with better uplink states, and the method without fairness may contribute to a catastrophic result that the D2D pairs with the poor uplink states are bankrupt in the condition of communication for an extended period. As a result, the performance of communication is restricted and degraded.

## 3. Resource Allocation Based on Weighted Efficiency Interference-Aware for D2D Underlaid Communication

In this section, the proposed algorithm is introduced, and the details are presented. The transmit power of D2D pairs are obtained, so to traverse all RBs to access the SINR of D2D pairs and CUs, D2D pairs are clustered by their SINR under the condition that the CUs can communicate regularly. The resource allocation is implemented according to the result obtained from clustering.

### 3.1. Distributed Transmit Power of D2D Pairs

Several factors influence the performance of D2D pairs and CUs in a communication system. Generally speaking, D2D pairs shall transmit signals whereas never disturb the QoS of CUs. It is assumed that CUs transmit signals with fixed power to reduce the complexity of the allocation algorithm. Moreover, given that the transmit power of D2D pairs is assumed to be changeable, and each D2D pair determines the transmit power by itself without coordination, conditionally. Inspired by [31], the maximum transmit power of D2D pairs is derived as

(11) $$\frac{p_{C\_j}d_{j,B}^{-\alpha }{\left|h_{j,B}\right|}^{2}}{\sum_{i=1}^{N}\beta_{i,j}^{CD}p_{D\_i}d_{i,B}^{-\alpha }{\left|h_{i,B}\right|}^{2}+N_{c}}\geq \gamma_{min}^{\left(C\right)}$$

Then, the maximum transmit power of D2D pairs can also be calculated by (11) as

(12) $$p_{D\_i}\leq p_{Di\_max}=\frac{p_{C\_j}d_{j,B}^{-\alpha }{\left|h_{j,B}\right|}^{2}/\gamma_{min}^{\left(C\right)}-N_{c}}{\sum_{i=1}^{N}\beta_{i,j}^{CD}d_{i,B}^{-\alpha }{\left|h_{i,B}\right|}^{2}}\;\mathrm{for}\;\forall j\in X$$

where, in (12), we use the fact that $\sum_{i=1}^{N}\beta_{i,j}^{CD}=1$, which will be explained henceforth, if the *j*-th CU shares the RB with the *i*-th D2D pair, and it means that there is only one unit in the summation. Thus, the maximum transmit power of D2D pairs can be denoted as

(13) $$p_{Di\_max}^{\prime }=min\left(p_{Di\_max}\;,\;p_{D\_max}\right)$$

After the session of D2D communication is set up, the link CSI and threshold $G_{min}^{D}$ are known by all receivers (D2D pairs and CUs), D2D transmitters use maximum transmit power $p_{Di\_max}^{\prime }$ with probability $P_{i}$ to transmit signals to associated receivers. It means that the maximum transmit power is used by D2D pairs as long as the uplink channel state is good enough. The mean transmit power of D2D pairs is given by

(14) $$\begin{array}{ll} E\left(p_{D\_i}\right) & =p_{Di\_\max}^{\prime }P_{i}\\ & =p_{Di\_\max}^{\prime }P\left[d_{i,i}^{-\alpha }{\left|h_{i,i}\right|}^{2}\geq G_{min}^{D}\right]\\ & =p_{Di\_\max}^{\prime }exp\left(-d_{i,i}^{\alpha }G_{min}^{D}\right) \end{array}$$

where the last equality is the result of a Rayleigh fading assumption. Recalling that the transmit power of each D2D pair is determined by itself as shown in (14), the path loss is generally fixed in a certain communication environment so that the transmit power of D2D pairs are affected by distance $d_{i,i}$ between transmitters and associated receivers and the selected threshold $G_{min}^{D}$. Transmit power would be small if either one of two parameters is large enough. That is, appropriate selection of threshold $G_{min}^{D}$ is important to the performance of the system.

### 3.2. Clustering of D2D Pairs

After traversing all RBs, the received SINR of D2D pairs for each RB and the SINR of CUs received at BS are calculated according to (4) and (5). D2D pairs are clustered if the performance of CUs is not disturbed, and the SINR of D2D pairs meet the particular demand. The number of clusters is assumed to be the same as that of RBs, and the clusters are denoted as $S_{j}^{C}$ $\left(j=1,2,\ldots ,M\right)$, which means that one cluster is an intended user set of one RB. All clusters are denoted as $S^{C}$ and $S^{C}=\left\{S_{j}^{C}\right\}$, and each D2D pair can be an intended user of all RBs as the conditions presented above are satisfied.

 **Definition 2.**  *All D2D pairs in a cluster are the intended users of one RB, which means that each D2D pair can reuse this RB and*$\underset{j\in X}{⋃}S_{j}^{C}\subseteq DP$.

 **Definition 3.**  *Each D2D pair can be an intended user of multiple RBs. Thus, one D2D pair can be found in different clusters indicated as*

(15) $$S_{j}^{C}\cap S_{k}^{C}\neq \emptyset \;\mathrm{for}\;\exists j,k\in X\;\mathrm{and}\;j\neq k$$

After clustering the D2D pairs, the result we identified is that a row vector is the available RBs of one D2D pair and a column vector is an intended user set of one RB if we apply a matrix to represent the allocation with D2D pairs as ordinate and RBs as abscissa. For each intended user set, the D2D pairs compete for each other for the qualification of reusing RB. To fairly compete among D2D pairs, we have the following definition.

 **Definition 4.**  *The weight of D2D pairs in each intended user set that expresses the qualification of sharing RBs is calculated by SINR ratio as*

(16) $$\omega \left(i|S_{j}^{C}\right)=\frac{\gamma_{D\_i}}{\sum_{\;S_{j}^{C}}\gamma_{D\_i}}$$

D2D pairs in each intended user set are weighted by their corresponding SINR accordingly. Nevertheless, one D2D pair in different intended user sets may have different weights due to the interference received from CUs are different. The value of weight satisfies $\omega \left(i\;|\;S_{j}^{C}\right)\in \left[0,1\right]$, as we consider that the D2D pair with higher weight in an intended user set has prior opportunity to share RB with CUs.

### 3.3. Resource Allocation Algorithm

Each available RB in the network needs to select a D2D pair in its intended user set to act as the actual user aimed to achieve a high system throughput performance. One intended user set may have multiple D2D pairs in accordance with the clustering rules, and some RBs have few intended users due to poor uplink states. To reduce the complexity of the algorithm, we have the following definition.

 **Definition 5.**  *Each row and column of the matching matrix*$\beta^{CD}$*have one ‘1’ at most after resource allocation, so the number of RBs allocated to a D2D pair is no more than one whilst one RB only can be reused by one D2D pair simultaneously in a scheduling period as well.*

(17) $$\sum_{i=1}^{N}\beta_{i,j}^{CD}\leq 1,\forall j\in X$$

(18) $$\sum_{j=1}^{M}\beta_{i,j}^{CD}\leq 1,\forall i\in Y$$

Accordingly, D2D pairs would never reuse the same RB and the interference between D2D pairs do not exist, that is $I_{dd}^{i}=0$. The actual user set of one cluster is denoted as $U_{j}^{D}$ and the number of D2D pairs in $U_{j}^{D}$ shall be no more than 1, according to the definition given as $\mathrm{card}\left(U_{j}^{D}\right)\leq 1$ and $U_{j}^{D}\subseteq S_{j}$ for ∀j∈X.

Thus, the optimization problem of system throughput in (6) can be rewritten as

(19) $$\max R_{i,j}^{B}$$

s.t. (7)–(10), (17) and (18).

 **Definition 6.**  *The efficient allocation that maximizes the system throughput is denoted as*$B^{*}=argmax\left(R_{i,j}^{B}\right)$.

D2D pairs share RBs with CUs to promote the system throughput on the premise that the performance of CUs is ensured to be normal. To describe the allocation result of D2D pairs and RBs intuitively, we have the example of allocation with *M* = 6 and *N* = 5 as

$$\beta^{CD}=\left[\begin{array}{cccccc} 0 & 1 & 0 & 0 & 0 & 0\\ 1 & 0 & 0 & 0 & 0 & 0\\ 0 & 0 & 0 & 1 & 0 & 0\\ 0 & 0 & 0 & 0 & 1 & 0\\ 0 & 0 & 1 & 0 & 0 & 0 \end{array}\right]$$

Recalling that corresponding receivers obtained the CSI of D2D pairs and CUs and fed back to the BS, the resource allocation is implemented based on the acceptable interference for both D2D pairs and CUs, which is accompanied by interference problem. The BS selects proper RBs to D2D pairs whenever SINR of D2D links and cellular links are satisfied. The details of resource allocation are shown in Table 1 as follows.

Note that, if weight value of D2D pairs is equal to 1 in some clusters, this signifies that these clusters have only one D2D pair, so the RBs corresponded to these clusters have only one intended user. In such a case, these clusters have priority to reuse RBs for fairness. Thus, steps 8 and 9 are implemented for clusters with one D2D pair.

From the steps of the algorithm shown above, we can note that the weight value of D2D pairs is not fixed in different allocation periods. If a D2D pair is chosen by one RB and eliminated in other intended user sets, the weight of the remaining D2D pairs in those intended user sets are changed in the next period. Thus, the allocation of RBs is implemented once in an allocation period and implemented *M* times at most for all RBs after allocation. The matching matrix that can denote the efficient allocation is obtained as well as the actual user set $U_{j}^{D}$ of each RB.

### 3.4. Complexity and Utility of Resource Allocation

Performance and complexity of an algorithm is a trade-off problem, as complexity indicates the occupation of system resources (time and memory) when executing the program of the algorithm. As illustrated, the path of the programming problem can be solved by the exhaustive search to obtain the optimal solution whereas accompanied to extremely high complexity. Each D2D pair in the communication system that consists of *N* D2D pairs and *M* RBs can be allocated with *M* possible results. Consequently, all D2D pairs are allocated with $M^{N}$ possible results, so the complexity of the exhaustive search is denoted by $O\left(M^{N}\right)$.

In the proposed WE-I-A algorithm, the weight value of D2D pairs needs to be calculated for several iterations. For one iteration, BS chooses the available RBs for D2D pairs as far as possible and the number of times calculated is $C_{M}^{1}+C_{M-1}^{1}+\ldots +C_{M-\left(N-1\right)}^{1}=MN-N\left(N-1\right)/2$. Without loss of generality, each D2D pair shall traverse the overall RBs, so the number of iterations is *N*. Definitively the complexity of the proposed algorithm is $O\left(MN^{2}-N^{2}\left(N-1\right)/2\right)$. It is obvious that $O\left(M^{N}\right)>\;O\left(MN^{2}-N^{2}\left(N-1\right)/2\right)$, so the complexity of the proposed algorithm is much lower.

To reveal the utility function of resource allocation, we have the following definition.

 **Definition 7.**  *The utility of resource allocation represents the integrated efficiency of the number of active D2D pairs and the throughput gain as*

(20) $$E=\frac{card\left(\beta^{CD}\right)\times R_{D2D}}{N\times R{\_}_{opt}}\;\mathrm{for}\;\beta_{i,j}^{CD}\in \beta^{CD}\subseteq B$$

where $R_{\_opt}$ is the optimal system throughput obtained by exhaustive search with a finite number of D2D pairs and CUs.

From the expression, as the number of D2D pairs (*N*) is determined, it has been revealed that the utility is not only related to the number of active D2D pairs ($\mathrm{card}\left(\beta^{CD}\right)$), but also to the throughput of both D2D pairs and the total system ($R_{D2D}$ and $R_{\_opt}$). The number of active D2D pairs can reflect the interference received by the cellular communication system to a certain degree.

The active D2D pairs are those which are able to communicate successfully by reusing RBs in a communication scenario, so the inactive ones are those that fail in the ability of communication, due to factors like low transmit power, poor uplink state, etc. The utility is an integrated parameter which is formulated to reveal the efficiency of resource allocation. The number of active D2D pairs and their throughput reflect the effect of the resource allocation algorithm in a specific communication environment.

## 4. Simulation Results and Analysis

In this section, we provide the numerical simulation results to illustrate the performance of the proposed WE-I-A algorithm, where relevant analysis and evaluation are conducted. Simulations using MATLAB are designed, and the main parameters are listed in Table 2. The path-loss model and shadow fading are considered for both cellular uplink users and D2D pairs. In order to demonstrate the generality of the experiment, simulation results are evaluated by averaging 500 independent experiments.

### 4.1. SINR

**Figure 4:**
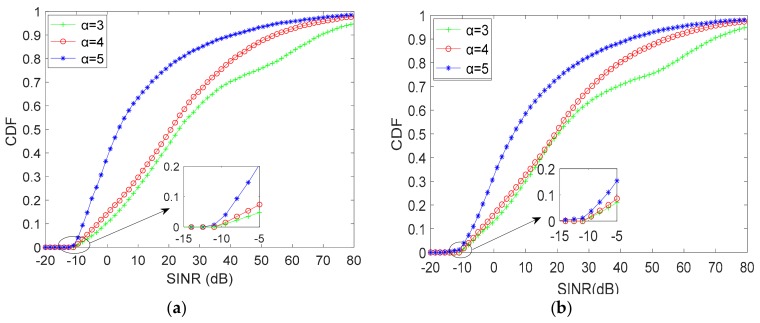
SINR of CUs for proposed WE-I-A algorithm: (**a**) *N* = 8, *M* = 10 and (**b**) *N* = 16, *M* = 20.

**Figure 5:**
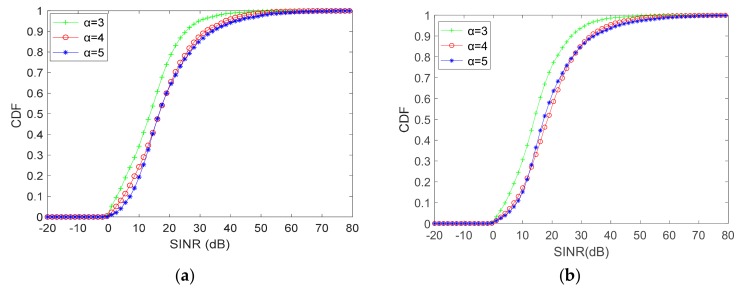
SINR of active D2D pairs for proposed WE-I-A algorithm: (**a**) *N* = 8, *M* = 10 and (**b**) *N* = 16, *M* = 20.

In this evaluation, the number of D2D pairs and RBs are assumed to be *N* = 8, *M* = 10 and *N* = 16, *M* = 20. For different uplink states, we inspect the performance of terminals (D2D pairs and CUs) and the differences between two scale of the communication systems. The SINR of CUs and active D2D pairs are illustrated in Figure 4 and Figure 5, respectively.

By observing (**a**) and (**b**) in Figure 4, we can note that the SINR of CUs is approximately inversely proportional to the path-loss exponent α. That is, the smaller the α, the better the SINR performance of the CUs. Moreover, as α is equal to 3 and 4, which means a good or normal uplink state, the cumulative distribution function (CDF) of SINR shows a tiny difference. It is clear that the SINR of α = 5 is different from that of α = 3 and 4. Nevertheless, when CUs share RBs with D2D pairs, the SINR of CUs are required to be larger than −10dB, the format requirements of modulation and coding scheme (MCS) would not satisfy the communication system if otherwise. As shown in Figure 4a,b, SINR of CUs less than −10 dB for the proposed algorithm are 1% to 4% in different uplink states that satisfies the requirements of communication. For the differences between different scale of the system, the scale has little influences on the performance of CUs when comparing these figures.

**Figure 6:**
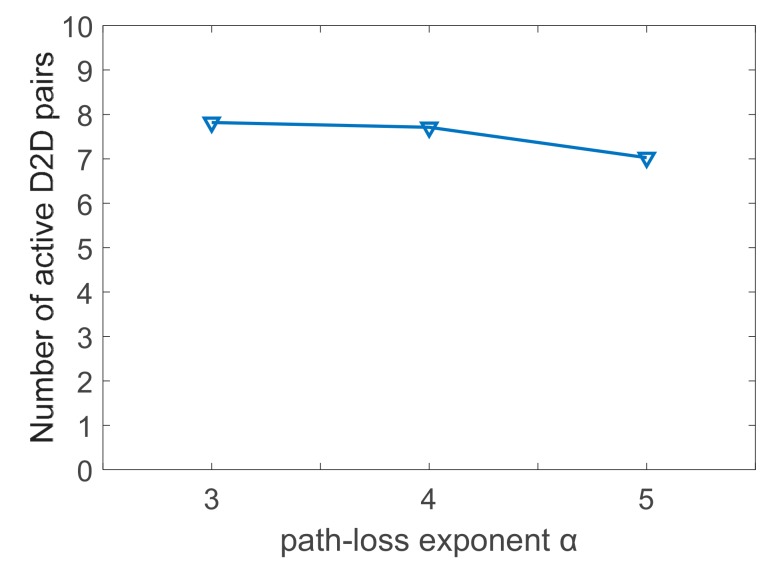
Number of active D2D pairs for proposed WE-I-A algorithm: *N* = 8, *M* = 10.

SINR of the proposed algorithm received at active D2D receivers is shown in Figure 5, where (a) is the scenario of *N* = 8, *M* = 10 and (b) is *N* = 16, *M* = 20, and the allowed minimum SINR at active D2D receivers is assumed to be 0dB. In these charts, we learn that the received SINR of D2D pairs are mainly less than 30dB. Then, SINR in the case of α = 3 is some lower than that of α = 4 and 5. Moreover, SINR of D2D pairs is shown little differences between α = 4 and 5. Therefore, even the uplink state is found to be poor, D2D pairs would perform well as a result of the short communication distance. As to the diverse scale of communication system, the comparison of D2D pairs’ performance between the case of (a) and (b) shows little differences as well. Thereafter, we can conclude that the scale of the system has little influences on the proposed algorithm noting the results obtained in Figure 4 and Figure 5. Furthermore, it does not signify that larger α can contribute to higher system throughput, and the reasons are twofold. The poor uplink state would decrease the SINR of CUs on one side, and some RBs cannot be reused under the constraints of the minimum SINR on the other side. The transmit power of D2D pairs would be limited in this communication environment. Based on this fact, the system throughput descends in the case of α = 5 due to the decreased number of active D2D pairs. As depicted in Figure 6 for the scenario of (a), the number of active D2D pairs in different uplink states for the situation of α = 3, α = 4, and α = 5 are about 7.8, 7.7, and 7.0, respectively.

### 4.2. Throughput

**Figure 7:**
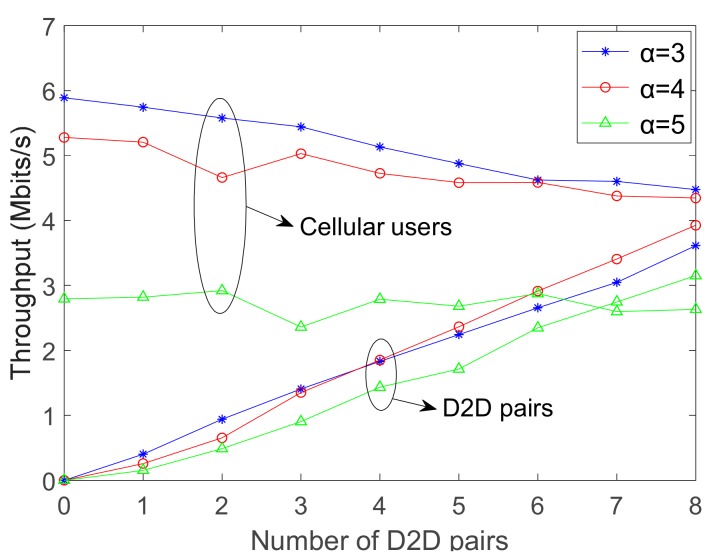
Throughput of D2D pairs in different uplink states for proposed WE-I-A algorithm.

As illustrated in Figure 7, the throughput of CUs and D2D pairs in different uplink states for proposed WE-I-A algorithm is shown. For CUs, we can see that the throughput decreases inversely with the ascending number of D2D pairs, and the situation is much more evident in the case of α = 3, since the CUs are easily influenced by D2D communication when the uplink state is good. Approaching α = 5, the throughput of CUs is much lower than that of α = 3 and α = 4, whereas the uptrend of throughput is much gentler.

It is noted that, the poor uplink state has been the significant effect factor of cellular communication rather than the interference caused by D2D pairs at that moment, so the throughput of D2D pairs in different uplink states increases with the ascending number of D2D pairs. Therefore, the performance of system throughput is advocated by proposed WE-I-A algorithm. Additionally, the throughput curve of D2D pairs with α = 3 is slightly larger when the abscissa is smaller, α = 4 becomes larger as increases in the abscissa. With the cellular system more sensitive in the case of α = 3, multiple D2D pairs can interfere the cellular communication. Conclusively, D2D communication is enhanced by the ascending number of D2D pairs.

From Figure 7, two essential points are observed. First, the uptrend (throughput) of D2D pairs is evident with the downtrend of CUs for each α, so D2D communication disturbs the performance of CUs within a certain degree but has a promotion to the whole system. Moreover, D2D communication would have few effects on CUs when the uplink state is weak, whereas leading to a considerable promotion for a communication system. Overall, the proposed WE-I-A algorithm can perform well even if the uplink state is found to be reduced.

**Figure 8:**
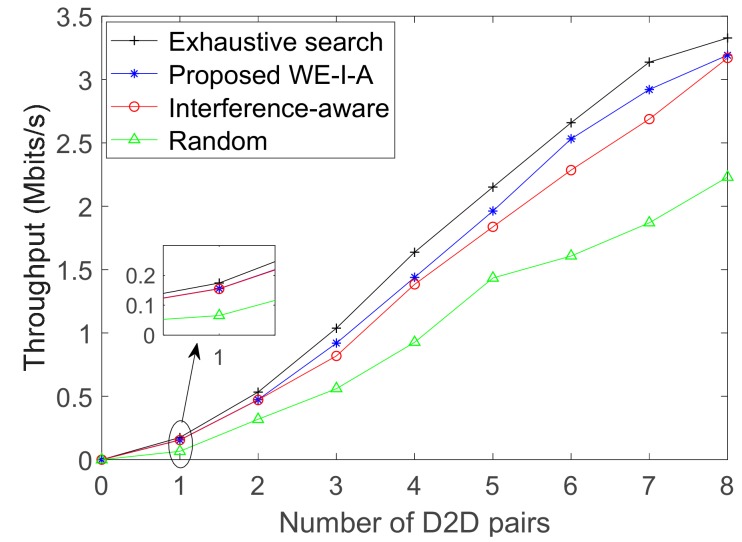
System throughput for different allocation algorithms in case of α = 5.

The system throughput for different allocation algorithms in the case of α = 5 is shown in Figure 8, the exhaustive search guarantees an upper bound of the system throughput, whereas the proposed WE-I-A algorithm is found to be a suboptimum method with much lower complexity than that of exhaustive search. Even though the interference-aware allocation is a classical algorithm as well as the random allocation [32,33], the proposed algorithm has superior performance over both algorithms, even in the case of the poor uplink state.

### 4.3. Utility of Allocation

**Figure 9:**
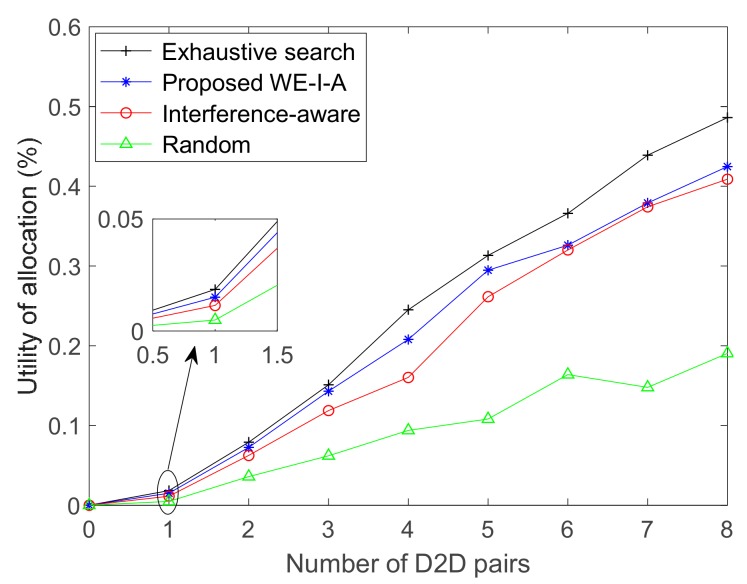
Utility of resource allocation for different allocation algorithms in case of α = 5.

Recalling that the proposed algorithm can perform well in the poor uplink state, Figure 9 illustrates the utility curve of different resource allocation algorithms in case of α = 5, where it is seen that, the utility is low when the number of D2D pairs is small, since CUs mainly contribute to the system throughput at that instant. The throughput of D2D pairs increases with the ascending number of D2D pairs, whereas CUs slightly decreases, as shown in Figure 7. Nevertheless, the utility gradually increases, it does not increase continuously due to the constraint on the finite number of RBs even with more D2D pairs. Therefore, the uptrend of the utility curve would slow down with the ascending number of D2D pairs.

## 5. Conclusions and Future Work

In this paper, we have investigated how to relieve the effects of interference between D2D pairs and CUs in the uplink of the D2D underlaid network to improve the system throughput. The weighted efficiency interference-aware algorithm is proposed as the mechanism to allocate the spectrum resources for D2D communication. The weight of each D2D pair in each intended user set is calculated, to provide precise details of the proposed allocation algorithm. Simulation results demonstrate that the SINR of cellular uplink users perform well when D2D pairs reuse the RBs even in the case of the poor uplink state. Meanwhile, the system throughput goes up with as the number of D2D pairs increases, since the throughput of D2D communication rises proportionally as that of CUs decreases. Furthermore, the proposed allocation algorithm is stable over different parameters of users and channel states, provides high utility and performs superiorly to interference-aware and random allocation.

As future work, the investigation on the situation that one D2D pair reuses multiple RBs of CUs to improve the efficiency of spectrum resource is aimed further. Also, to apply and adapt the proposed algorithm in industrial production and real cases, such as smart agriculture and smart mine, where the communication conditions and environments are highly yet dynamically varied.

## Figures and Tables

**Table 1:** Proposed WE-I-A algorithm.

Input: Output:	$\mathrm{System}\;\mathrm{Area}\;R,\;\mathrm{Transmit}\;\mathrm{Power}\;\mathrm{of}\;\mathrm{CUs}\;p_{c\_j}$ $\mathrm{Actual}\;\mathrm{User}\;\mathrm{Set}\;U_{j}^{D}$
1:	Initialize, $\gamma_{min}^{\left(D\right)}$, $\gamma_{min}^{\left(C\right)}$, $G_{min}^{D}$, and $\beta_{i,j}^{CD}\;=\;1$,∀i∈Y, ∀j∈X
2:	Calculate $p_{D\_i}$ by (14)
3:	Obtain $\gamma_{D\_i}$ and $\gamma_{C\_j}$ by (4) and (5)
4:	Set $\beta_{i,j}^{CD}$ to 0 if one of (7) and (8) is not satisfied
5:	Obtain $S_{j}^{C}$ from $\beta^{CD}$
6:	Calculate $\omega \left(i\;|\;S_{j}^{C}\right)$ by (16)
7:	**For** each j∈X do
8:	**If**$\omega \left(i^{*}\;|\;S_{j^{*}}^{C}\right)\;=\;1$, then
9:	Keep $\beta_{i^{*},j^{*}}^{CD}\;=\;1$, then go to 13.
10: 11:	**Else** Select the D2D pair with maximum weight to be an actual user of $\;S_{j^{*}}^{C}$ and keep $\beta_{i^{*},j^{*}}^{CD}\;=\;1$
12:	**End if**
13:	Set the other position of i^*^-th row and j^*^-th column of $\beta^{CD}$ to 0
14:	**End for**
15:	Obtain $U_{j}^{D}$ from $\beta^{CD}$
16:	Return $U_{j}^{D}$

**Table 2:** Main simulation parameters.

Parameters	Value
System area	R = 500 m
Transmit power of CUs	50 mW
Maximum transmit power of D2D	1 mW
Noise spectral density	−174 dBm/Hz
Maximum transmit distance of D2D	50 m
Sub-channel bandwidth	100 kHz
Minimum SINR of the cellular link	−10 dB
Minimum SINR of D2D link	0 dB
